# Supplementary material for: Behavioural inference from signal processing using animal-borne multi-sensor loggers: a novel solution to extend the knowledge of sea turtle ecology
Source: R Soc Open Sci. 2020 May 13;7(5):200139. doi: 10.1098/rsos.200139 (PMC7277266; doi:10.1098/rsos.200139)
Supplement: Supplementary Tables and Figure [file rsos200139supp2.pdf]

SUPPLEMENTARY

Supp. Table 1. Summary of immature green turtle measures and video recorded time.

Individual number	Carapace Length (cm)	Carapace Width (cm)	Mass (kg)	Video Recorded Time (h)	Date
#1	44	3.2	10	1,5	14/02/2018
#2	55	43.2	37	5,9	20/05/2018
#3	83	73	68	4,0	22/05/2018
#4	80	77	64	3,8	26/05/2018
#5	84	74.8	63.8	3,9	04/10/2018
#6	88,3	74.8	83	6,6	06/10/2018
#7	82	78	67	8,0	08/10/2018
#8	72.7	67.9	59	1,0	10/10/2018
#9	43.3	38.3	9.5	4,8	11/02/2019
#10	83.5	77.2	70	2,2	11/02/2019
#11	94.2	82	99.8	7,8	18/02/2019
#12	100.3	89.5	109	2,2	08/04/2019
#13	79.5	75.4	67.6	14,6	13/05/2019

Supp. Table 2: Definition and associated category of the behaviour observed from the video-recorder data and associated with a unique acceleration-gyroscope-depth signal.

Category	Behaviour	Definition
**Breathing**		Turtle is breathing one time at the surface
**Feeding**	Catching	Turtle is catching a floating element in water
	Catching jellyfish	Turtle is catching a jellyfish
	Chewing on movement	Turtle is chewing seagrass or algae and keeps swimming
	Chewing stationary	Turtle is chewing seagrass or algae landing at the bottom
	Chewing jellyfish	Turtle is chewing a jellyfish
	Grabbing on movement	Turtle is grabbing seagrass or algae at the bottom and keeps swimming
	Grabbing stationary	Turtle is grabbing seagrass or algae landing at the bottom
	Grabbing the wall	Turtle is grabbing seagrass or algae from a wall or a rock
**Gliding**	Gliding ascent	Turtle is swimming passively without flipper beat toward the surface
	Gliding descent	Turtle is swimming passively without flipper beat toward the bottom
**Resting**	Resting	Turtle is resting at the bottom without moving
	Resting active	Turtle is resting at the bottom but it is agitated and moves the head
	Resting in flow	Turtle is resting in the flow leading to passive body movement
	Resting watching	Turtle is resting at the bottom watching behind
**Scratching**	Scratching	Turtle is scratching its shell
	Scratching head	Turtle is scratching its head

	Scratching camera	Turtle is scratching and the camera touches the rock or other element
**Staying at the surface**		Turtle is breathing several time at the surface
**Swimming**	Stepping back	Turtle is moving backward at the bottom
	Swimming 1 ascent	Turtle is swimming toward the surface using one front flipper
	Swimming 1 descent	Turtle is swimming toward the bottom using one front flipper
	Swimming 1 horizontally	Turtle is swimming horizontally using one front flipper
	Swimming ascent	Turtle is swimming toward the surface
	Swimming descent	Turtle is swimming toward the bottom
	Swimming horizontally	Turtle is swimming horizontally
	Swimming fast ascent	Turtle is swimming fast toward the surface
	Swimming fast descent	Turtle is swimming fast toward the bottom
	Swimming fast horizontally	Turtle is swimming fast horizontally
	Swimming in place	Turtle is swimming but stays in place
	Swimming on the sea floor	Turtle is moving landing at the bottom
	Prospection	Turtle is swimming slowly prospecting on the right and on the left
	Watching	Turtle is swimming watching behind
	Left U-turn	Turtle is turning on the left
	Right U-turn	Turtle is turning on the right
**Unknown**	Escape	Turtle is escaping to avoid aggression from another turtle.
	Flipper beat	Turtle is scratching its head with its flipper
	Foraging	Turtle is touching the bottom to search for something
	Interaction	Turtle is touching another turtle with its beak
	Landing	Turtle is abruptly landing at the bottom
	Obstacle	Turtle is taking an obstacle
	Pursuit	Turtle is pursuing a second turtle
	Regurgitating	Turtle is regurgitating
	Sand	Turtle is on the ground and sending the sand up with its front flippers
	Shaking	Turtle is shaking
	Shaking head	Turtle is shaking its head
	Hunting jellyfish	Turtle is modifying its trajectory to catch a jellyfish

Supp. Table 3. Difference between the total times predicted and the actual (video-based) ones for each behavioural category in seconds, in percent of the total individual observed time and in percent of the behavioural expressed time for each immature green turtles.

Behaviour	#1			#2			#3			#4			#5			#6			#7		
	s	%t*	%b**	s	%t*	%b**	s	%t*	%b**	s	%t*	%b**	s	%t*	%b**	s	%t*	%b**	s	%t*	%b**
Breathing	6,7	0,1	18,8	101,4	0,5	33,7	25,6	0,2	69,9	27,8	0,2	137,6	47,2	0,3	71,3	30,3	0,1	34,7	70,2	0,2	79,0
Feeding	0,9	0,0	-	31,7	0,2	2,1	78,6	0,6	48,6	110,5	0,8	20,5	56,1	0,4	36,9	1006,2	4,2	51,5	279,4	1,0	401,4
Gliding	0,2	0,0	-	4,4	0,0	0,5	101,4	0,7	27,7	340,8	2,5	65,1	100,0	0,7	47,4	51,6	0,2	18,2	26,1	0,1	2,5
Other	0,0	0,0	-	124,2	0,6	48,2	123,8	0,9	73,3	73,6	0,5	26,0	123,7	0,9	83,8	530,9	2,2	64,9	31,2	0,1	22,9
Resting	4,1	0,1	-	15,3	0,1	0,2	261,2	1,8	3,4	270,8	2,0	5,7	101,1	0,7	1,7	517,7	2,2	4,6	1640,6	5,7	8,4
Scratching	0,7	0,0	-	26,7	0,1	5,2	50,2	0,4	8,7	225,3	1,7	12,6	150,5	1,1	1880,7	33,1	0,1	3,7	223,5	0,8	164,3
Stay in surface	0,0	0,0	-	271,9	1,3	30,3	42,0	0,3	3,0	172,6	1,3	11,2	465,2	3,3	33,4	133,3	0,6	5,2	237,9	0,8	8,1
Swimming	342,2	6,4	6,5	85,3	0,4	1,3	31,4	0,2	0,8	449,9	3,3	11,0	562,1	4,0	8,8	600,3	2,5	10,0	757,2	2,6	15,8
Transition	4,4	0,1	-	68,0	0,3	-	24,3	0,2	-	28,6	0,2	-	44,9	0,3	-	124,8	0,5	-	55,3	0,2	-

Behaviour	#8			#9			#10			#11			#12			#13			Average		
	s	%t*	%b**	s	%t*	%b**	s	%t*	%b**	s	%t*	%b**	s	%t*	%b**	s	%t*	%b**	s	%t*	%b**
Breathing	2,15	0,1	37	21,7	0,1	37,8	10,8	0,1	39,7	2,8	0	2,118	23,8	0,1	31,818	47,3	0,1	16,1	32,131	0,2	46,87
Feeding	4,796	0,1	-	391	2,3	37,9	261,7	3,2	39,6	72,34	0,3	1130	156,6	0,8	74,617	246,1	0,5	138	207,38	1,1	180,1
Gliding	3,32	0,1	3,3	60,1	0,3	4,78	108,7	1,3	84,3	491,2	1,8	80,69	51,7	0,3	6,7874	2899	5,5	128	326,02	1	39,06
Other	27,93	0,8	62	251	1,5	96,3	107,1	1,3	41,1	151,1	0,5	64,86	192,5	1	74,449	121,9	0,2	64,9	143,02	0,8	60,2
Resting	36,8	1,1	5,2	1169	6,8	16,3	51,89	0,6	8,62	51,44	0,2	0,293	1058	5,5	8,4318	4527	8,6	16,5	746,53	2,7	6,601
Scratching	28,3	0,8	-	108	0,6	169	196	2,4	942	51,68	0,2	29,27	187,2	1	95,889	258,2	0,5	118	118,43	0,7	311,8
Stay in surface	1,45	0	0,3	54,4	0,3	5,27	65,9	0,8	8,06	140,6	0,5	9,471	98,5	0,5	7,4934	272,8	0,5	8,4	150,5	0,8	10,84
Swimming	27,82	0,8	1,4	678	3,9	10,7	35,74	0,4	0,65	457,3	1,6	5,91	366,7	1,9	9,6364	1342	2,5	7,1	441,28	2,4	6,884
Transition	10,82	0,3	-	29,5	0,2	-	14,24	0,2	-	16,02	0,1	-	85,45	0,4	-	115	0,2	-	47,789	0,2	-

* in percent of the total individual observed time

** in percent of the behavioural observed time

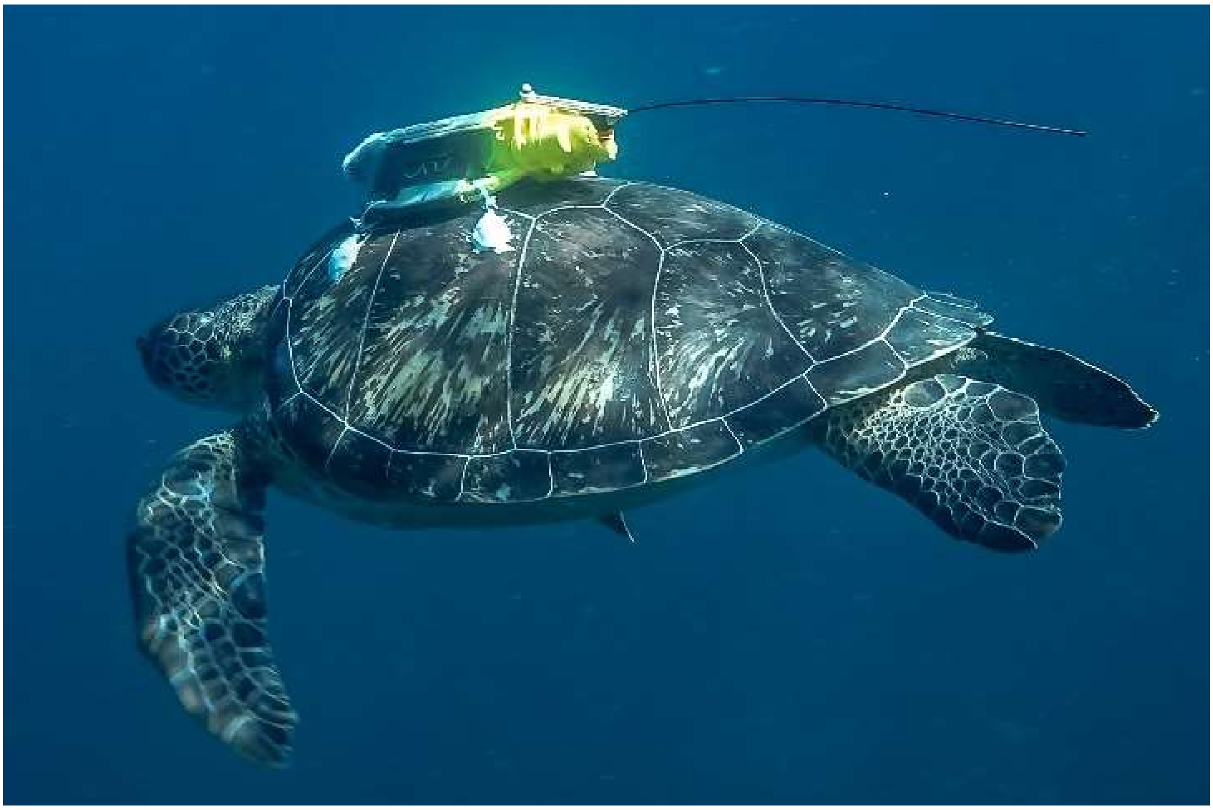

Supp. Figure 1. Free-ranging immature green turtle equipped with a device combining a high-frequency tri-axial accelerometer, a high-frequency tri-axial gyroscope, a depth recorder and video-recorder.
